# Review and statistical analysis of clinical management of feline leishmaniosis caused by *Leishmania infantum*

**DOI:** 10.1186/s13071-022-05369-6

**Published:** 2022-07-11

**Authors:** Maria Garcia-Torres, María Cristina López, Séverine Tasker, Michael Rex Lappin, Carles Blasi-Brugué, Xavier Roura

**Affiliations:** 1 Hospital Clínic Veterinari, Universitat Autònoma de Barcelona, Barcelona, Spain; 2 Bristol Veterinary School, University of Bristol, Langford, Bristol, UK; 3 Linnaeus Veterinary Limited, Shirley, Solihull UK; 4 Department of Clinical Sciences, Colorado State University, Fort Collins, USA

**Keywords:** Cats, Leishmaniasis, Serology, Allopurinol, Practitioners

## Abstract

**Background:**

There is limited information about feline leishmaniosis (FeL) management in clinical practice. *Leishmania infantum* is the species of *Leishmania* most frequently reported in both dogs and cats in countries of the Mediterranean region (henceforth ‘Mediterranean countries’), Central and South America, and Iran. This study was conducted to provide veterinary clinicians with an updated overview of evidence-based information on leishmaniosis in cats.

**Methods:**

A review was performed using PubMed, Science Direct, Google Scholar and Web of Science. Case reports of FeL caused by *L. infantum* were sought for the period 1912 to 1 June 2021.

**Results:**

Sixty-three case reports are included in this review. Fifty-nine out of the 63 cats were from Europe, mostly from Mediterranean countries (88.9%). Most of them were domestic short-haired cats (90%) with a mean age of 7.9 years, and had access to the outdoors (77.3%). Sixty-six percent of the cats had comorbidities, of which feline immunodeficiency virus infection was the most frequent (37.7%). Dermatological lesions (69.8%) was the most frequent clinical sign, and hyperproteinemia (46.3%) the most frequent clinicopathological abnormality. Serology was the most performed diagnostic method (76.2%) and was positive for 93.7% of cats. Medical treatment was applied in 71.4% of cats, and allopurinol was the most used drug (74.4%). Survival time was greater for treated cats (520 days; 71.4% of cats) than non-treated cats (210 days; 25.4%).

**Conclusions:**

The majority of the cats had comorbidities, of which feline immunodeficiency virus was the most frequent. Dermatological lesions were frequently reported, and systemic clinical signs and clinicopathological abnormalities were also common. Serology may be useful for the diagnosis of FeL in clinical practice, and a positive titer of ≥ 1/40 may be a useful cut-off for sick cats. The reported treatments and dosages varied, but there was a good clinical response and longer survival in most of the cats treated with allopurinol monotherapy.

**Graphical abstract:**

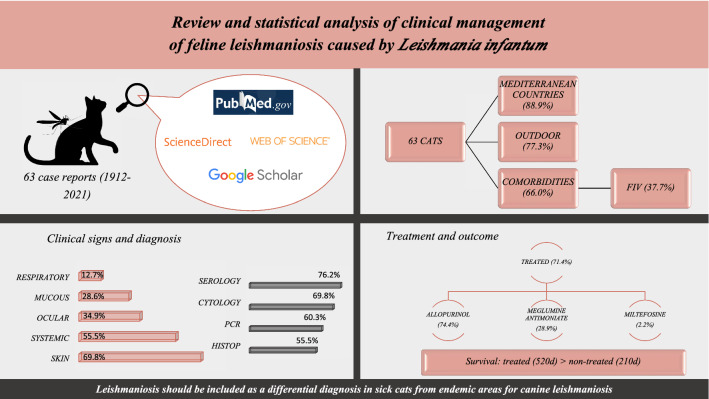

## Background

Leishmaniosis is a zoonotic vector-borne disease with a worldwide distribution. The causal agents of leishmaniosis are intracellular protozoans of the genus *Leishmania*, which are transmitted by female phlebotomine sand flies. Although dogs are regarded as the main reservoir host, during the last decades feline leishmaniosis (FeL) has gained more attention from veterinary practitioners and researchers in areas endemic for leishmaniosis. Although the number of cats with leishmaniosis is currently considered negligible in endemic areas, a high percentage of cats test positive for the disease [serology, polymerase chain reaction (PCR), or both] [1–7]. Several *Leishmania* spp. can infect cats (*Leishmania infantum*, *Leishmania mexicana*, *Leishmania venezuelensis*, *Leishmania tropica, Leishmania major, Leishmania amazonensis*, and *Leishmania braziliensis*), and *L. infantum* is the species most frequently reported in both dogs and cats in countries of the Mediterranean region (henceforth ‘Mediterranean countries’), Central and South America, notably Brazil, and Iran [8–14].

Although it is likely that the first case of *L. infantum* infection in a cat was that described in 1912 by Sergent et al. [15], the number of case reports of FeL has been increasing globally, especially in the last 30 years [7, 16–56]. However, compared to canine leishmaniosis (CanL), there is still limited information on the clinical management of FeL. Furthermore, much of the available information on FeL is not specific to *L. infantum* infection, and is mostly from reports providing little scientific evidence, such as descriptive case series, isolated case reports, extrapolations from CanL studies, or those based on the personal experience of respected experts, whilst few are based on recent research in cats [8–11, 55, 57–59]. Moreover, few of the published research studies describe the clinical management of leishmaniosis in cats, and instead focus on the epidemiology and prevalence of leishmaniosis in cats in regions that are endemic or non-endemic for CanL [1, 4, 5, 13, 57, 60–82].

 The following are crucial for the management of FeL, especially within the current context of the lack of clinical guidelines for this disease: understanding how leishmaniosis caused by *L. infantum* affects cats; identifying the most useful diagnostic tests and most effective treatments; and determining the prognostic factors and expected prognosis. We conducted a review to assess the risk factors, clinical signs and clinicopathological alterations, diagnostic methods, treatment, and outcome of all known published cases of FeL, to provide veterinary clinicians with an updated overview of this disease.

## Methods

### Search strategy

Independent literature searches were conducted between March and August 2021 by two of the authors (MGT and XR) using the databases and keywords listed in Table 1. When there were potential discrepancies between the selected articles, a third author (MCL) participated in the final decision. Additional studies were identified by contacting the authors of the publications, and by searching the publications’ reference lists. First, the titles and abstracts of all the articles identified in the searches were evaluated, and then the full texts of those considered potentially relevant were examined thoroughly.

**Table 1:** Search strategy

Source	Index terms
PubMed (https://pubmed.ncbi.nlm.nih.gov)	Feline leishmaniosis OR feline leishmaniasis OR cat, *Leishmania* OR feline, *Leishmania infantum* OR feline, case series OR feline, case reports
Science Direct (https://www.sciencedirect.com/)	
Google Scholar (https://scholar.google.com)	
Web of Science (https://apps.webofknowledge.com/)	

### Inclusion and exclusion criteria

The inclusion criteria were as follows: feline case reports or case series of FeL caused by *L. infantum* from 1912 to 1 June 2021, including signalment, a description of clinical signs, diagnostic methods, treatment protocols and outcome for each cat. The exclusion criteria were as follows: duplicate records, case reports of leishmaniosis caused by *Leishmania* species other than *L. infantum*, studies that contained information that was confusing or not sufficiently comprehensible for analysis, and reviews or meta-analyses that did not provide specific data on the factors that had been investigated for each cat. Data on treatment and outcome were not available for some of the included cases. The case reports selected in this way were included in a Microsoft Excel database and duplicate data were eliminated. The final collated publications were used for the statistical analysis, for which data from each of the included studies were extracted.

### Data extraction

A pre-established protocol was used to extract the following data: when and where the research was carried out (year, country and geographic region), signalment, clinical presentation, breed, sex, age, indoor/outdoor, comorbidities, clinical signs, clinicopathological alterations, diagnostic method, treatment, and outcome.

### Statistical analysis

Continuous variables were summarized by mean, range, and SD. For association and risk factor analysis for infection, geographic data were grouped into Mediterranean and non-Mediterranean countries; breed data were grouped into domestic short haired (DSH) and non-DSH (Siamese, Siberian, crossbreed, unknown breeds); lifestyle was divided into indoor or outdoor; and clinical signs were grouped into cutaneous, mucocutaneous, ocular, respiratory and systemic. To group clinical signs, we followed the classification used by the clinicians who authored each case report; lymphadenomegaly, fever, lethargy, poor body condition, pallor, hepato-splenomegaly, weight loss and abdominal distension were considered systemic signs. Comorbidities were defined as diseases other than FeL, other medical conditions, prescribed medication, and pathogens that could modify the immune response

In the statistical evaluation of diagnostic method reliability, cytology and/or histopathology were considered as gold standards for the direct identification of parasites in accordance with experts’ recommendations for FeL. An indirect fluorescent antibody technique (IFAT) had been used in the majority of cases, so to enable the statistical analysis of positive quantitative serological results, all the data were transformed according to the same titer scale based on World Organization for Animal Health recommendations [83] and divided into titer ranges of 1/40 to ≤ 1/80, > 1/80 to ≤ 1/160, > 1/160 to ≤ 1/320, > 1/320 to ≤ 1/640, and > 1/640. For those cases where quantitative serologic data were not supplied, and only a positive or negative result from a test such as western blot, the results were grouped as qualitative.

 All the statistical analyses were performed using IBM SPSS version 20 for Mac. Univariate analyses were performed, and the results are presented as the number of affected cats in relation to the total number of cats for which the finding was described. Data were evaluated for normal distribution using a Kolmogorov–Smirnov test. In the univariate analyses, when only two independent continuous variables were compared, an independent-sample* t*-test was performed according to the data distribution. For the categorical variables, a chi-square test of association was used, and for paired samples the Wilcoxon test was used. SPSS was used to calculate the expected frequencies. The rule used was that, at most, only 20% of expected frequencies should be less than 5. The Pearson correlation (*r*) test of association was used for survival and age analyses. A Kaplan–Meier survival analysis with log ranks was used to test for significant differences between survival curves for treatment applied. The treatments were grouped as follows: (i) allopurinol, (ii) allopurinol plus meglumine antimoniate, (iii) allopurinol plus miltefosine, (iv) meglumine antimoniate, (v) other, and (vi) no treatment. When survival data were not exactly defined, the highest known values were used as the survival data for the analyses. *P* < 0.05 was considered the critical level of significance.

## Results

### Case selection

**Fig. 1:**
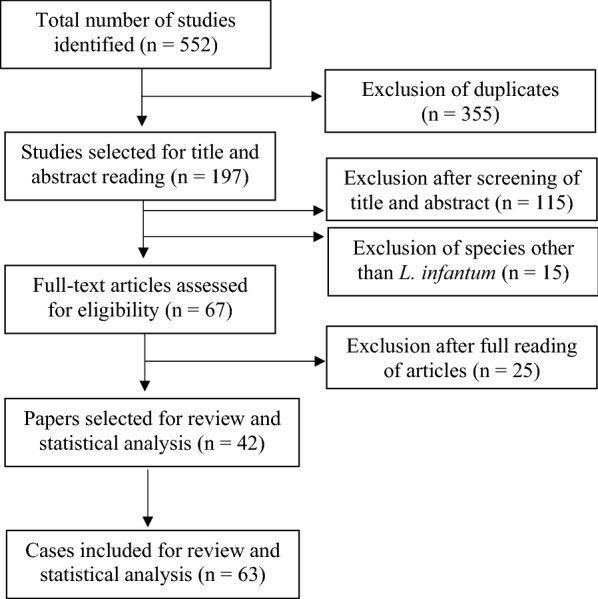
Flow chart showing the search and selection process for the inclusion of articles in this study

The online literature search identified 552 potentially relevant publications. A total of 355 duplicate publications were excluded. After the initial screening of the data, based on title and/or abstract evaluation, another 130 publications were excluded. A further 25 were excluded during a second selection process based on the full-text evaluation of the 67 remaining publications. A total of 42 articles (63 cats) were finally found to be eligible for inclusion in this review and the data subjected to statistical analysis (Fig. 1; Table 2).

**Table 2:** Cases included in the statistical analysis according to continent, country, type of study and number of cats described

Continent	Country	Type of study (*n*)	No. of cats	References
Europe	Italy	Case reports (6), case series (1), systematic review (1)	16	[7, 26, 27, 32, 36, 44, 49, 55]
	France	Case reports (6)	6	[19, 21, 22, 29, 39, 40]
	Spain	Case reports (13), case series (1)	24	[23–25, 31, 33, 34, 37, 38, 46, 51–54, 56]
	Portugal	Case reports (6), case series (1)	9	[20, 35, 42, 43, 45, 48, 50]
	Switzerland	Case report (1), case series (1)	3	[30, 41]
	Cyprus	Case report (1)	1	[47]
Africa	Algeria	Case report (1)	1	[17]
	Reunion Island	Case report (1)	1	[18]
South America	Brazil	Case report (1)	1	[28]
Asia	Vietnam	Case report (1)	1	[16]

### Geographic region

All the cases were of domestic cats living in Europe (59), Brazil (1), Vietnam (1), Reunion Island (1), and Algeria (1). The European cases were from Spain (24), Italy (16), Portugal (9), France (6), Switzerland (3), and Cyprus (1). There was a statistically significant association between location and the number of cases, with a higher prevalence in Mediterranean (56/63, 88.9%) compared to non-Mediterranean (7/63, 11.1%) countries (χ^2^ = 38.111, *df* = 1, *P* < 0.001) (Table 3).

**Table 3:** Univariate analysis of the association between geographic region, breed, sex, and lifestyle of cats with leishmaniosis

Covariate	Levels	*n* (%)	*χ*^2^ (*df*)	*P*-value
Geographic region	Non-Mediterranean	7/63 (11.1)		
	Mediterranean	56/63 (88.9)	38.111 (1)	< 0.001*
Breed	Siamese	4/63 (6.3)		
	Siberian	1/63 (1.6)		
	Crossbreed	1/63 (1.6)		
	Unknown	3/63 (4.8)		
	DSH	54/63 (85.7)	170.571 (4)	< 0.001*
Sex	Male	29/62 (46.7)		
	Female	33/62 (53.2)	0.258 (1)	0.611
Lifestyle	Indoor	5/22 (22.7)		
	Outdoor	17/22 (77.3)	6.545 (1)	< 0.011*

*Mediterranean* Mediterranean region, *DSH* domestic short haired

*P < 0.05

### Signalment

The age of the cats at clinical presentation was known in 56/63 cases, and ranged from 2 to 21 years (mean 7.9 ± 4.1 years). Breed was described for 60 out of the 63 cases, and was as follows: 54 DSH (85.7%), four Siamese (6.3%), one Siberian (1.6%) and one crossbreed (1.6%) cat. DSH were more likely to be infected than non-DSH breeds (χ^2^ = 170.571, *df* = 4, *P* < 0.001). Sex was reported in 62 out of 63 cases, and there was a slightly higher prevalence in females (53.2%) than in males (46.7%), although this difference was not statistically significant (χ^2^ = 0.258, *df* = 1, *P* = 0.611). Lifestyle was known for 22 cats, of which 17 were outdoor (77.3%) and five indoor cats (22.7%). There was an association between an outdoor lifestyle and infection (χ^2^ = 6.545, *df* = 1, *P* ≤ 0.011) (Table 3).

Information on comorbidity status was available for 53 cats (Table 4); of these 35 (66.0%) had comorbidities and 18 (34.0%) did not. Of the 35 cats with comorbidities, 22 (62.9%) had only one comorbidity, whereas 13 (37.1%) had two or more. Positive feline immunodeficiency virus (FIV) antibody status was the most prevalent comorbidity, but the association between this and leishmaniosis was not statistically significant (χ^2^ = 0.277, *df* = 1, *P* = 0.599).

**Table 4:** Comorbidities in cats with leishmaniosis

Comorbidities	No. of cats (from a total of 53)	%
Present	35	66.0
Absent	18	34.0
FIV +ve	20	37.7
FIV −ve	33	62.3
FeLV +ve	4	7.5
FeLV −ve	49	92.5
Corticosteroid treatment		
Yes	11	20.8
No	42	79.2
Other medical conditions and pathogens		
* Bartonella henselae*	2	3.8
* Candidatus* Mycoplasma haemominutum	2	3.8
Feline coronavirus	3	5.6
* Toxoplasma* spp.	4	7.5
* Hepatozoon* spp.	1	1.9
Pemphigus	1	1.9
Pregnancy	1	1.9
Squamous cell carcinoma	2	3.8
Diabetes mellitus	1	1.9
Epidermoid carcinoma	1	1.9

*FIV* Feline immunodeficiency virus,* FeLV* feline leukemia virus

### Clinical presentation

The clinical signs and lesions that were reported are given in Table 5. The most frequent clinical signs were cutaneous (44/63; 69.8%), followed by systemic (35/63; 55.5%), ocular (22/63; 34.9%), mucocutaneous (18/63; 28.6%) and respiratory (8/63; 12.7%). Many of the cats showed a combination of clinical signs (37/63; 58.7%). There was a statistically significant association between dermatological signs and FIV (χ^2^ = 7.185, *df* = 1, *P* = 0.007), and between ocular signs and the neutered status of females (χ^2^ = 17.814, *df* = 3, *P* < 0.001). However, no other statistically significant associations were found between groups of clinical signs and age, sex, breed, or comorbidities (Table 6). Lymph node size was described for 45/63 cats from physical examination; the percentage of cats with lymph nodes of normal size (27/45; 60.0%) was greater than that of cats with lymphadenomegaly (18/45; 40.0%).

**Table 5:** Clinical signs and lesions described in cats (*n* = 63) with leishmaniosis

Clinical signs and lesions	Frequency	
	No. of cats	%
Cutaneous	44	69.8
Ulcerative dermatitis	20	31.7
Nodular dermatitis	14	22.2
Alopecia	9	14.3
Desquamative dermatitis	7	11.1
Crusty dermatitis	5	7.9
Pruritus	2	3.1
Bloody cyst	2	3.1
Papular dermatitis	1	1.6
Systemic	35	55.5
Lymph node enlargement	18	28.5
Anorexia/hyperorexia	14	22.2
Weight loss	14	22.2
Depression	13	20.6
Fever	7	11.1
Pallor	5	7.9
Vomiting/diarrhea	3	4.7
Polyuria/polydipsia	1	1.6
Icterus	1	1.6
Ocular	22	34.9
Uveitis	13	20.6
Conjunctivitis	7	11.1
Nodular blepharitis	6	9.5
Ulcerative blepharitis	3	4.7
Keratoconjunctivitis	1	1.6
Ulcerative keratitis	1	1.6
Mucocutaneous	18	28.6
Stomatitis/gingivostomatitis	11	17.4
Glossitis	3	4.7
Nasal ulcers	3	4.7
Nasal pustules	2	3.1
Nasal depigmentation	1	1.6
Oral ulcers	1	1.6
Respiratory	8	12.7
Nasal discharge	4	6.3
Stridor	3	4.7
Stertor	1	1.6
Sneezing	1	1.6
Reverse sneezing	1	1.6
Dyspnea	1	1.6
Bronchitis	1	1.6

**Table 6:** Analysis of association (χ^2^) between sex, age, breed, comorbidities (any), FIV, FeLV, and steroid treatment with groups of clinical signs

Variable	Cutaneous	Mucocutaneous	Ocular	Respiratory	Systemic
Sex	χ^2^ = 6.065, *df* = 3 *P* = 0.108	χ^2^ = 0.386, *df* = 3, *P* = 0.943	χ^2^ = 17.814, *df* = 3, *P* = < 0.001*	χ^2^ = 3.285, *df* = 3, *P* = 0.350	χ^2^ = 1.444, *df* = 3, *P* = 0.695
Age	*t*_(63)_ = 1.206, *P* = 0.233	*t*_(63)_ = 1.525, *P* = 0.133	*t*_(63)_ = 1.906, *P* = 0.062	*t*_(63)_ = 0.370, *P* = 0.713	*t*_(63)_ = 0.912, *P* = 0.366
Breed	χ^2^ = 4.534, *df* = 4, *P* = 0.339	χ^2^ = 4.200, *df* = 4, *P* = 0.380	χ^2^ = 3.230, *df* = 4, *P* = 0.520	χ^2^ = 8.126, *df* = 4, *P* = 0.087	χ^2^ = 2.663, *df* = 4, *P* = 0.616
Comorbidities	χ^2^ = 1.914, *df* = 1, *P* = 0.167	χ^2^ = 0.005, *df* = 1, *P* = 0.945	χ^2^ = 0.015, *df* = 1, *P* = 0.901	χ^2^ = 0.105, *df* = 1, *P* = 0.746	χ^2^ = 0.010, *df* = 1, *P* = 0.922
FIV	χ^2^ = 7.185, *df* = 1, *P* = 0.007*	χ^2^ = 1.150, *df* = 1, *P* = 0.283	χ^2^ = 0.070, *df* = 1, *P* = 0.791	χ^2^ = 0.090, *df* = 1, *P* = 0.764	χ^2^ = 1.539, *df* = 1, *P* = 0.215
FeLV	χ^2^ = 0.638, *df* = 1, *P* = 0.424	χ^2^ = 0.155, *df* = 1, *P* = 0.694	χ^2^ = 0.277, *df* = 1, *P* = 0.599	χ^2^ = 0.658, *df* = 1, *P* = 0.417	χ^2^ = 1.002, *df* = 1, *P* = 0.317
Steroid treatment	χ^2^ = 0.117, *df* = 1, *P* = 0.732	χ^2^ = 2.622, *df* = 1, *P* = 0.105	χ^2^ = 0.011, *df* = 1, *P* = 0.916	χ^2^ = 0.300, *df* = 1, *P* = 0.584	χ^2^ = 0.895, *df* = 1, *P* = 0.344

For abbreviations, see Table 4

*P < 0.05

When clinicopathological abnormalities were reported, hyperproteinemia was the most frequent (19/41; 46.3%), followed by anemia (16/48; 33.3%), neutrophilia (9/48, 18%), thrombocytopenia (8/48, 16.6%), proteinuria (7/46; 15.2%) and azotemia (7/47; 14.9%). Hypergammaglobulinemia was the most frequent alteration detected by serum protein electrophoresis (27/38; 71.0%) followed by hypoalbuminemia (9/47; 19.1%). Other reported clinicopathological alterations were neutropenia (4/48, 8.3%), eosinophilia (3/48, 6.25%), pancytopenia (1/48, 2%) and an increase in alanine transaminase level (2/48, 4.1%).

### Diagnostic methods

Cytology, for the detection of* Leishmania* amastigotes, was the most common first line or preferred diagnostic option of practitioners for the diagnosis of FeL (28/63, 44.4%), followed by histopathology (20/63, 31.7%), serology (17/63, 26.9%) and PCR (3/63, 4.7%). There was no statistically significant association (χ^2^ = 8.980, *df* = 2, *P* = 0.062) between results from the PCR and those from cytological and/or histopathological examination (widely used as confirmatory tests for FeL and considered gold standards here). However, a statistically significant association between cytology and/or histopathology and seropositivity (χ^2^ = 26.913, *df* = 14, *P* = 0.020) was found.

Of the complete diagnostic procedures (both first line and additional diagnostic tests), antibody detection techniques were performed most (48/63; 76.2%), and comprised IFAT (28/48), qualitative serology (11/48), enzyme-linked immunosorbent assay (5/48), and direct agglutination (4/48). Antibody tests were positive for 45/48 cats (93.7%) and negative for 3/48 (6.3%); there was a statistically significant association between *L. infantum* antibody positive status and diagnosis of FeL by cytology and/or histopathology (χ^2^ = 36.750, *df* = 2, *P* < 0.001). In cats seropositive according to quantitative tests (35/45, 77.8%), the titers ranged from 1/40 to ≤ 1/80 in 5/35 (14.3%), from > 1/80 to ≤ 1/160 in 3/35 (8.6%), from > 1/160 to ≤ 1/320 in 5/35 (14.3%), from > 1/320 to ≤ 1/640 in 5/35 (14.3%), and were > 1/640 in 17/35 cats (48.5%). No statistically significant association was found between serological titers and category of clinical signs or clinicopathological abnormalities (*P* > 0.05). Qualitative positive serology was reported for 10/45 cats (22.2%).

Polymerase chain reaction (using blood, lymph node, bone marrow, spleen tissue; ocular, lung or skin samples) was performed for 38 of the 63 cats (60.3%), and was positive for 36 of them (94.7%) (Table 7). Cytology was performed for 44/63 cats (69.8%) and histopathology for 35/63 cats (55.5%) using different types of tissue. Cytology and histopathology were positive for *Leishmania* amastigotes in 39/44 cats (88.6%) and 31/35 (88.6%), respectively (Tables 8, 9). With respect to serological titers, no statistically significant association was found between PCR, cytology or histopathology with any category of clinical sign or clinicopathological abnormality (*P* > 0.05).

**Table 7:** Frequency of tested tissues and positive results from polymerase chain reaction (*PCR*)

Tested tissue	PCR (*n* = 38)	PCR +ve (*n* = 36, 94.7%)
Blood	18/38, 47.3%	16/18, 88.8%
Skin samples	6/38, 15.7%	6/6, 100%
Unknown origin	6/38, 15.7%	6/6, 100%
Lymph node	4/38, 10.5%	4/4, 100%
Bone marrow	4/38, 10.5%	4/4, 100%
Ocular samples	2/38, 5.2%	2/2, 100%
Spleen	2/38, 5.2%	2/2, 100%
Respiratory samples	1/38, 2.6%	1/1, 100%

**Table 8:** Frequency of tested tissues and parasite detection using cytology

Tested tissue	Cytology (*n* = 44)	Cytology +ve (*n* = 39, 88.6%)
Skin lesions	20/44, 45.4%	18/20, 90%
Lymph node	18/44, 40.9%	16/18, 88.8%
Ocular lesions	7/44, 15.9%	7/7, 100%
Bone marrow	6/44, 13.6%	5/6, 83.3%
Spleen	2/44, 4.5%	2/2, 100%
Liver	1/44, 2.2%	1/1, 100%
Blood	1/44, 2.2%	1/1, 100%
Respiratory lesions	1/44, 2.2%	1/1, 100%

**Table 9:** Frequency of tested tissues and parasite detection using histopathology

Tested tissue	Histopathology (*n* = 35)	Histopathology +ve (*n* = 31, 88.6%)
Skin lesions	22/35, 62.8%	20/22, 90.9%
Ocular lesions	5/35, 14.2%	4/5, 80%
Respiratory lesions	4/35, 11.4%	3/4, 75%
Oral lesions	2/35, 5.7%	2/2, 100%
Bone marrow	2/35, 5.7%	1/2, 50%
Spleen	2/35, 5.7%	2/2, 100%
Kidney	1/35, 2.8%	1/1, 100%

### Treatment

Medical treatment was administered in 45/63 cats (71.4%); 16/63 (25.4%) did not receive any treatment, and treatment was not stipulated for 2/63 (3.2%). Allopurinol was used in 37/45 cats (74.4%), followed by meglumine antimoniate in 13/45 (28.9%) and miltefosine in only one cat of the 45 (2.2%). Although the dosages varied, the most frequent were 10 mg/kg twice a day (BID) per os (PO) for at least 6 months for allopurinol (20/37 cats; 54.0%), 50 mg/kg once a day (SID) subcutaneously (SC) for 30 days for meglumine antimoniate (5/13 cats; 38.5%), and 2 mg/kg SID PO for 28 days for miltefosine in the only cat in which it was used. Allopurinol was used as monotherapy in 28/37 cats (75.7%) and in combination with meglumine antimoniate or miltefosine in 8/37 (21.6%) and 1/37 (2.7%) cats, respectively. Meglumine antimoniate was used as monotherapy in 5/13 cats (38.5%) and in combination with allopurinol in 8/13 (61.5%). Adverse effects associated with treatment were reported in 10/45 cats (22.2%), and were mainly associated with allopurinol (7/10), and affected the kidney (4/10), skin (2/10) and liver (1/10).

### Outcome

**Fig. 2:**
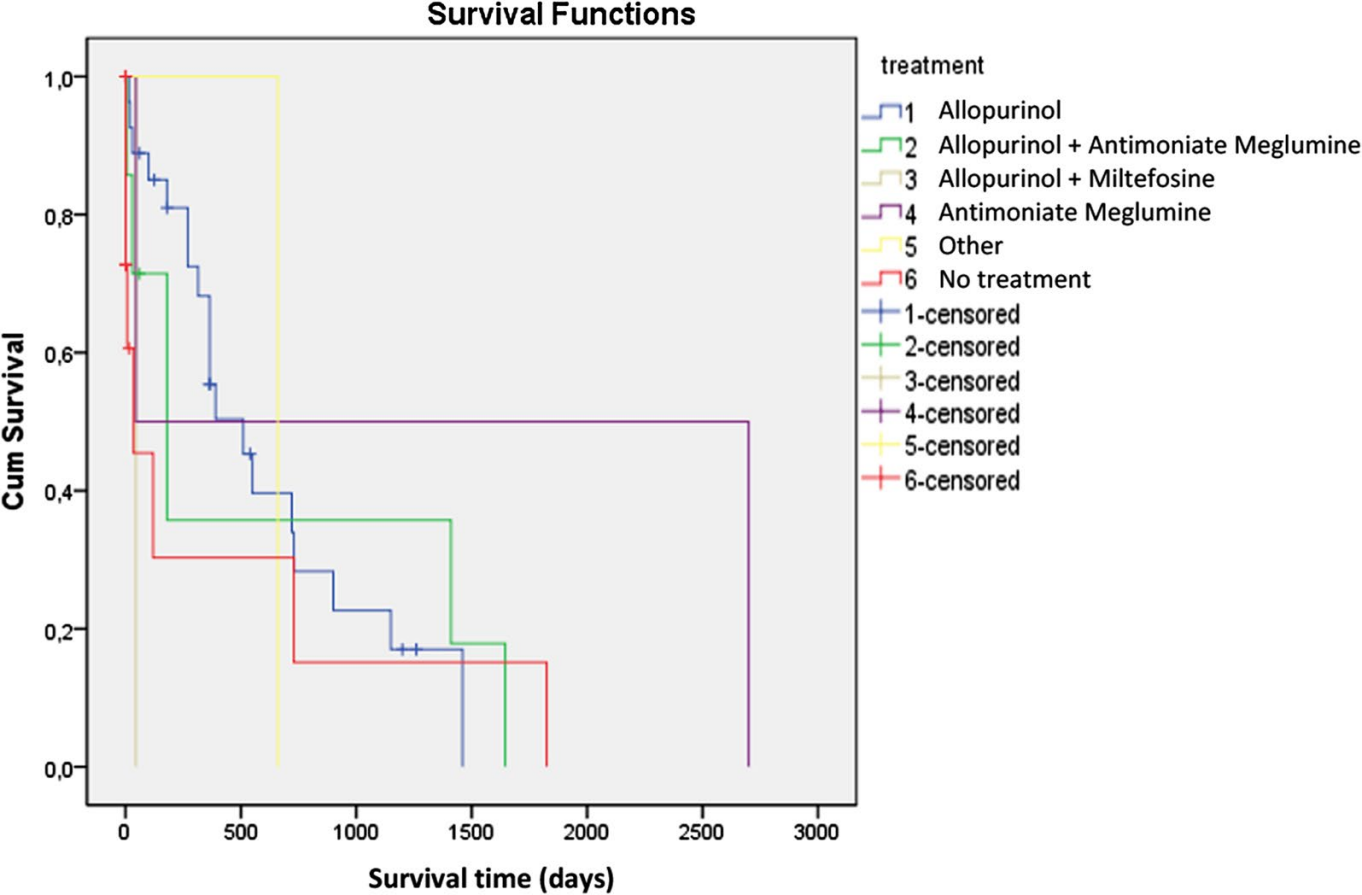
Kaplan–Meier survival curves of cats treated with allopurinol, allopurinol plus meglumine antimoniate, allopurinol plus miltefosine, meglumine antimoniate, other, or no treatment. Median survival time of the treated cats was as follows: allopurinol (28/63; 619 days); allopurinol plus meglumine antimoniate (8/63; 614 days); allopurinol plus miltefosine (1/63; 45 days); meglumine antimoniate (5/63; 1372 days); other such as fluconazole, metronidazole, spiramycin or lomidine (3/63; 660 days); no treatment (10/63; 411 days)

Survival time ranged from 0 to 2700 days, with a mean of 432 days (± 575). Mean survival time was significantly longer for treated cats than non-treated cats (520 days and 210 days, respectively) (χ^2^ = 15.311, *df* = 1, *P* = 0.002) (Fig. 2). However, no significant association was found between survival time and any other variable (Table 10).

**Table 10:** Analysis of association (χ^2^) between treatment, age, breed, sex, lifestyle, comorbidities (any), clinical signs (any), clinicopathological abnormalities (any), PCR positive test, serological titer and survival

Variable	Survival
Treatment (yes/no)	χ^2^ = 15.311, *df* = 1, *P* = 0.002*
Age	*r*_(63)_ = -0.028, *P* = 0.856
Breed	χ^2^ = 17.165, *df* = 4, *P* = 0.144
Sex	χ^2^ = 3.112, *df* = 3, *P* = 0.960
Lifestyle	χ^2^ = 0.889, *df* = 1, *P* = 0.889
Comorbidities (any)	χ^2^ = 0.381, *df* = 1, *P* = 0.944
Cutaneous clinical signs	χ^2^ = 1.693, *df* = 1, *P* = 0.638
Mucocutaneous clinical signs	χ^2^ = 3.100, *df* = 1, *P* = 0.377
Ocular clinical signs	χ^2^ = 3.742, *df* = 1, *P* = 0.291
Respiratory clinical signs	χ^2^ = 1.098, *df* = 1, *P* = 0.778
Systemic clinical signs	χ^2^ = 6.014, *df* = 1, *P* = 0.111
Anemia	χ^2^ = 0.669, *df* = 1, *P* = 0.881
Hyperproteinemia	χ^2^ = 1.048, *df* = 1, *P* = 0.790
Hyperglobulinemia	χ^2^ = 1.495, *df* = 1, *P* = 0.683
Hypergammaglobulinemia	χ^2^ = 2.252, *df* = 1, *P* = 0.522
Hypoalbuminemia	χ^2^ = 1.927, *df* = 1, *P* = 0.588
Azotemia	χ^2^ = 5.555, *df* = 1, *P* = 0.135
Proteinuria	χ^2^ = 2.233, *df* = 1, *P* = 0.526
PCR +ve	χ^2^ = 9.932, *df* = 2, *P* = 0.128
Serological titer	χ^2^ = 18.920, *df* = 7, *P* = 0.590

*P < 0.05

## Discussion

The results of this study indicate that leishmaniosis should be included in the differential diagnosis of sick cats living in, or with a history of travel to, areas where CanL is endemic [1–3, 84]. Prevalences of FeL in endemic areas as shown by positive PCR range from 0 to 100% (mean 21.3%), whilst those indicated by positive serology range from 0 to 70.5% (mean 13.7%) [1, 10, 85]. Prevalences determined by both of these types of tests are lower for cats than dogs (63% and 27% for PCR and serology, respectively) [85]. There are also fewer clinical feline cases than canine cases in the literature [8–11, 59]. Thus, all this suggests that the prevalence of FeL could be about half that of CanL for the same geographical areas [60, 63, 74, 79–81]. Furthermore, as previously reported for CanL [86, 87], increased movement of pet cats between countries, especially inside Europe, could lead to clinical cases of FeL being diagnosed in areas that are not endemic for *L. infantum* [30, 41]. However, although there have been epidemiological studies on FeL in areas that are also endemic for CanL, such as Brazil [1, 84], only one case from Brazil was included in the current study. Possible reasons for this include the non-detection of other cases from Brazil due to the criteria used in this study, and/or perhaps because feline medicine is less developed there, and/or because most knowledge on leishmaniosis is focused on dogs. Thus, even in areas that are non-endemic for *L. infantum*, leishmaniosis should be considered as a differential diagnosis in cats with clinical signs or pathological alterations consistent with this disease.

Few epidemiological studies have reported significant associations between *L. infantum* infection in cats and their access to outdoors, that they are male, or their age when they are adults [60, 64]. Most of the cats affected by leishmaniosis in the present study were DSH with outdoor access, and had a mean age of 7.9 years. In contrast to other publications [60, 65, 66], we found that more female cats were diagnosed with leishmaniosis than male cats, although this difference was not statistically significant.

It is well recognized that susceptibility to progressive *Leishmania* infection and the development of clinical signs in dogs is mostly linked to an imbalance in the adaptive immune response, and probably associated with a predominant Th2 response and an impaired Th1 immune response [88]. However, in contrast to CanL, to the best of our knowledge, no prospective controlled studies have been published on immune mechanisms involved in the pathogenesis of FeL. Some investigations have suggested that cats may have a better immune response against *L. infantum* because the Th2 response plays a protective role [81, 89], or because there are other factors in seropositive cats that can control the development of patent leishmaniosis [90], such as the production of higher levels of interferon gamma, which plays a direct role in the regulation of Th1 cell development [91]. Thus, *Leishmania* spp. infection in cats might be more common than associated disease, and cats might be more resistant to disease development than dogs [9, 59]. Cats that develop leishmaniosis are often suspected of having impaired immunity because of comorbidities [8–11]. However, to date, a significant association has only been reported between *L. infantum* and FIV co-infection [67, 69, 70, 82, 92], although the results of other studies contradict this association [71–73, 81]. In this review, a high percentage (66.0%) of evaluated cats with leishmaniosis had comorbidities which are associated with potentially impaired immune competence, such as previous corticosteroid treatment, diabetes mellitus, epidermoid carcinoma, squamous cell carcinoma, pemphigus, or co-infections such as FIV, feline leukemia virus, *Bartonella henselae*, ‘*Candidatus* Mycoplasma haemominutum,’ feline coronavirus, *Toxoplasma* spp. and *Hepatozoon* spp. Furthermore, the current study confirms that FIV, which was detected in 37.7% of cats, is the most frequent comorbidity associated with FeL, although the association was not statistically significant.

Leishmaniosis in dogs has a wide range of clinical signs [8, 93–95], but extrapolating these to cats could mean that only the clinical signs of an infected cat that resemble those of CanL would be used in a differential diagnosis for FeL. This could lead to the misdiagnosis of FeL and thus the underestimation of its clinical relevance. The clinical signs of FeL described in this review are mainly dermatological (69.8%), followed by systemic (55.5%), ocular (34.9%), mucocutaneous (28.6%), and respiratory (12.7%). These results agree with those of previous studies, where dermatological lesions and systemic clinical signs, including lymph node enlargement, were reported frequently in cats infected with *L. infantum* [9, 10, 53]. These findings support the idea that, when caused by *L. infantum*, CanL and FeL present similarly, and thus FeL should also be included in the differential diagnosis when a wide range of systemic clinical signs of leishmaniosis present alone or in combination in a cat. Less frequent and/or severe, isolated clinical presentations may go unreported or misdiagnosed, and presumably could lead to underestimation of the clinical relevance of FeL [8]. Although multiple factors have been found to be statistically associated with a wide range of clinical manifestations in FeL [66, 96], this review only found a statistically significant association between dermatological signs and FIV positive status. A potential explanation for this is that dermatological lesions associated with leishmaniosis and immunodeficiency are very similar in cats, so they could have been secondary to either condition [55].

In comparison to CanL, there is limited information about clinicopathological abnormalities associated with *L. infantum* infection in cats [41, 57, 97]. Of the cases analyzed here, hyperproteinemia (46.3%) was the most frequent laboratory abnormality, and hypergammaglobulinemia (71.0%) followed by hypoalbuminemia (19.1%) were the most frequent alterations seen on serum protein electrophoresis, in agreement with the results of two studies [41, 57], but in contrast with those of another study [58]. The percentages of cats presenting with neutrophilia, thrombocytopenia, proteinuria, and azotemia (18%, 16.6%, 15.2% and 14.9%, respectively) reported in the present study suggest that leishmaniosis should be a differential diagnosis for hypergammaglobulinemic cats with any of these conditions. However, although gamma globulin levels were significantly elevated in FeL, they could not be used to differentiate FeL from other inflammatory, neoplastic or vector-borne diseases in cats [97]. Furthermore, we report here the important finding that, in FeL, unlike in CanL, azotemia and proteinuria are frequently concurrent [98–100]. Although in dogs leishmaniosis is an important cause of proteinuria, this is not thought to be the case in cats [57, 58]. However, the present study shows that proteinuria is not that infrequent in cats with FeL caused by *L. infantum*, which suggests that it is important to perform urinalysis and measure the urinary protein to creatinine ratio to help in the early detection, and subsequent management, of chronic kidney disease in cats with leishmaniosis [101].

In contrast to CanL, there are fewer clearly recommended diagnostic tests for FeL [8–11]. Previous reviews and expert opinions suggest that, in clinical practice, the best means of confirming FeL is the detection of *Leishmania* amastigotes by cytology and/or histopathology, or the detection of *Leishmania* DNA by PCR; all of these tests can be performed using a sample of any type of affected tissue, including lymph node tissue, bone marrow or blood [8–10, 102, 103]. In agreement with published recommendations, cytology was found to be the preferred first line diagnostic technique for FeL, followed by histopathology, serology, and PCR. Furthermore, *Leishmania* DNA was easily detected by PCR, as were amastigotes by cytology and histopathology (i.e. in 94.7%, 88.6%, and 88.6% of cats with clinical leishmaniosis, respectively).

Conversely, serology has been suggested as being less useful for cats than for dogs for the diagnosis of leishmaniosis in clinical practice, and thus it may be more useful as an additional test to support the diagnosis of leishmaniosis and the follow up of sick cats with the disease [8–11]. Anti-*Leishmania* antibody detection techniques such as IFAT, enzyme-linked immunosorbent assay, direct agglutination and western blot have been extensively used in a wide range of studies on cats [1, 4, 5, 10, 57, 60, 62–64, 66–68, 70–78, 80–82]. However, the low levels of antibodies produced in cats due to their differing immune responses [74, 90] as compared to dogs, and/or the fact that few laboratories offer validated serological tests for FeL (compared to serological tests for CanL) may explain why serology has previously been overlooked as a diagnostic test for FeL in clinical practice [5, 57, 60, 62–64, 68–72]. For these reasons, it has been recommended that serologic results should be interpreted with caution and in combination with other diagnostic test results and the assessment of clinical signs for the diagnosis of FeL [8–11, 58]. Although a cut-off titer of 1:80 or above is considered adequate to discriminate between infected and non-infected cats [57, 64, 70, 74], the level of this titer is still controversial. In the current study, among first line and additional diagnostic tests, serology (73%) was the diagnostic technique most used by practitioners (positive serology in 93.5% of the sick cats), and positive serological results showed a statistically significant association with cytology and/or histopathology results. Furthermore, the positive seroreactivity titer range was wide (1/40 to > 1/640) in sick cats. However, no statistically significant association was found between serological titers and any type of clinical sign or clinicopathological abnormality, showing that there is no apparent direct relationship between clinical alterations and serological titers. Thus, we suggest that serology may be more useful as an initial diagnostic test than previously thought when cats that are suspected of having leishmaniosis are evaluated in clinical practice, and that a serological titer of ≥ 1/40 could be a suitable cut-off for the diagnosis of leishmaniosis caused by *L. infantum* in cats with clinical signs or clinicopathological alterations compatible with this disease. However, further studies are needed to confirm the best serological positive cut-off titer for the diagnosis of patent FeL.

An empirical therapeutic approach to FeL usually involves the extrapolation of recommendations for CanL [94, 104] as no studies have been conducted to evaluate the efficacy and safety of treatments in cats [8–11]. In line with the recommendations for CanL, the long-term administration of allopurinol, used as monotherapy (75.7%), or in combination with meglumine antimoniate (21.6%) or miltefosine (2.7%), was the drug of choice used for the treatment of 74.4% of the cats, followed by meglumine antimoniate and miltefosine for 28.9% and 2.2%, respectively. Adverse effects associated with treatment were reported in 22.2% of the cats [30, 48, 51, 53–55]. Although recent reports indicate that allopurinol can have different adverse effects in cats [48, 49] compared to those observed in dogs [94, 104], it should be noted that both spontaneous [105] and allopurinol-induced [51, 54] xanthinuria and urolithiasis have also been described in cats, hence it is recommended that urinalysis be performed during the treatment of FeL with allopurinol, as is done for CanL. Finally, propylene glycol is one of the excipients in the oral formulation of miltefosine that is licensed for the treatment of CanL, and has been reported to potentially cause a decrease in the lifespan of feline red blood cells due to the formation of Heinz bodies [8, 106]. Thus, the use of miltefosine should possibly be avoided in cats, or at least only used with caution until more scientific evidence on its effects have been published.

Survival time ranged from 0 to 2,700 days, with a mean of 432 days. Mean survival time was statistically greater for treated cats than non-treated cats (520 and 210 days, respectively), which supports previous recommendations that cats with clinical signs of leishmaniosis should be treated [8–10]. No statistically significant difference in survival time was found between treatment with allopurinol as monotherapy and allopurinol in combination with other drugs. This suggests that monotherapy with allopurinol could be used as a first line treatment in cats for at least for 6 months, and for those cats not responding to treatment with allopurinol alone, meglumine antimoniate could be added to try and further improve the clinical signs of leishmaniosis. However, caution is warranted because the effects of specific leishmaniosis treatments on survival could not be assessed here due to the great variability in the dosages of the different drugs used, and because the type, quality and intensity of supportive treatments used for each of the cats included in this study were unknown. No other statistically significant association was found between survival time and any other variable such as age, breed, sex, indoor/outdoor access, comorbidities, clinical signs or clinicopathological abnormalities, diagnostic test results and type of treatment, and thus no potential predictive factors for the prognosis of sick cats diagnosed with leishmaniosis were identified.

This study had several limitations, including (i) the risk of bias associated with the lack of inclusion of more cases due to the search criteria used, (ii) difficulties associated with the statistical analysis due to the fact that most of the included publications described a single clinical case, (iii) the heterogeneity of data due to different clinical management among years, (iv) imprecision regarding treatment effects due to the variability in dosages amongst the included cases, and (v) the lack of a comprehensive data set due to the retrospective nature of this study.

## Conclusions

The case reports of FeL caused by *L. infantum* included in the present study showed that the cats often had a comorbidity, with FIV infection being the most frequent of these. Dermatological alterations were the most frequently reported clinical sign in FeL caused by *L. infantum*, although systemic clinical signs and clinicopathological abnormalities, alone or in combination, were also common. Leishmaniosis should be included as a differential diagnosis for sick cats who live in, or have traveled from, areas endemic for CanL. The results of this study also indicate that serology could be useful as a first line diagnostic test for FeL, and that a positive titer of ≥ 1/40 could be a suitable cut-off for the diagnosis of leishmaniosis in sick cats that are suspected of having the disease. Finally, despite the limitations of this study, we found that there was good clinical response and prolonged survival in many cats given allopurinol as a monotherapy at 10 mg/kg PO BID for at least 6 months.

## Data Availability

Not applicable.

## Abbreviations

CanL: Canine leishmaniosis
DSH: Domestic short haired
FeL: Feline leishmaniosis
FIV: Feline immunodeficiency virus
IFAT: Indirect fluorescent antibody technique
PCR: Polymerase chain reaction
